# Altered neural encoding of vowels in noise does not affect behavioral vowel discrimination in gerbils with age-related hearing loss

**DOI:** 10.3389/fnins.2023.1238941

**Published:** 2023-11-14

**Authors:** Amarins N. Heeringa, Carolin Jüchter, Rainer Beutelmann, Georg M. Klump, Christine Köppl

**Affiliations:** Research Centre Neurosensory Science and Cluster of Excellence “Hearing4all”, Department of Neuroscience, School of Medicine and Health Science, Carl von Ossietzky University Oldenburg, Oldenburg, Germany

**Keywords:** presbycusis, consonant-vowel-consonant logatomes, Mongolian gerbil, speech-in-noise perception, temporal coding

## Abstract

**Introduction:**

Understanding speech in a noisy environment, as opposed to speech in quiet, becomes increasingly more difficult with increasing age. Using the quiet-aged gerbil, we studied the effects of aging on speech-in-noise processing. Specifically, behavioral vowel discrimination and the encoding of these vowels by single auditory-nerve fibers were compared, to elucidate some of the underlying mechanisms of age-related speech-in-noise perception deficits.

**Methods:**

Young-adult and quiet-aged Mongolian gerbils, of either sex, were trained to discriminate a deviant naturally-spoken vowel in a sequence of vowel standards against a speech-like background noise. In addition, we recorded responses from single auditory-nerve fibers of young-adult and quiet-aged gerbils while presenting the same speech stimuli.

**Results:**

Behavioral vowel discrimination was not significantly affected by aging. For both young-adult and quiet-aged gerbils, the behavioral discrimination between /eː/ and /iː/ was more difficult to make than /eː/ vs. /aː/ or /iː/ vs. /aː/, as evidenced by longer response times and lower d’ values. In young-adults, spike timing-based vowel discrimination agreed with the behavioral vowel discrimination, while in quiet-aged gerbils it did not. Paradoxically, discrimination between vowels based on temporal responses was enhanced in aged gerbils for all vowel comparisons. Representation schemes, based on the spectrum of the inter-spike interval histogram, revealed stronger encoding of both the fundamental and the lower formant frequencies in fibers of quiet-aged gerbils, but no qualitative changes in vowel encoding. Elevated thresholds in combination with a fixed stimulus level, i.e., lower sensation levels of the stimuli for old individuals, can explain the enhanced temporal coding of the vowels in noise.

**Discussion:**

These results suggest that the altered auditory-nerve discrimination metrics in old gerbils may mask age-related deterioration in the central (auditory) system to the extent that behavioral vowel discrimination matches that of the young adults.

## 1. Introduction

As we age, our auditory system inevitably degrades. By the age of 70, the number of sensory hair cells and auditory nerve (AN) fibers is approximately half of what it was at birth (Wu et al., 2020) and 63% of people over 70 experience some degree of hearing loss (Lin et al., 2011). One of the major consequences of age-related hearing loss is difficulty with communication, putting the elderly at risk for social isolation and depression. Even in elderly with clinically mild hearing loss, almost 60% reported communication difficulties (Dalton et al., 2003). In particular, understanding speech in a noisy environment, as opposed to speech in quiet, is strongly degraded with increasing age (Dubno et al., 1984; Fogerty et al., 2012; Füllgrabe et al., 2015). Therefore, it is crucial to understand the underlying mechanisms for age-related deficits in speech-in-noise perception.

There are multiple levels at which communication difficulties can be studied, ranging from the inability to distinguish between different meaningless syllables (logatomes) up to increased cognitive effort while listening to speech in noise. Here, we focus on the fundamental discrimination between vowels. Vowels differ from each other at the spectral level. Specifically, different vowels have different formant frequencies (*f_1_* – *f_3_*), which are global regions of maximum energy in the vowel spectrum (Diehl, 2008). Especially *f_1_* and *f_2_* were shown to be important for the discrimination of different vowels, both in gerbils and in humans (Peterson and Barney, 1952; Jüchter et al., 2022). The spectrum of spoken vowels, as opposed to that of whispered vowels, also comprises harmonics of the fundamental frequency (*f_0_*), the pitch of the speaker’s voice, which does not systematically differ between vowels (Diehl, 2008).

When presenting vowels in noise, both the *f_0_* and the formant frequencies are encoded by the AN in the exact spike timing, given the signal-to-noise ratio (SNR) is sufficiently high. This can be studied in recordings from single fibers of the AN and visualized in representation schemes based on metrics of spike timing, such as the averaged localized interval rate (ALIR) and the dominant component scheme (Young and Sachs, 1979; Sachs and Young, 1980; Delgutte and Kiang, 1984a). Not only for synthetic vowels in quiet, but also for naturally-spoken vowels presented in background noise do the formant frequencies appear in these spike-timing based representation schemes (Heeringa and Köppl, 2022). Specifically, inter-spike interval histograms were constructed to account for small changes in periodicity of *f_0_* over time, which is common for naturally spoken vowels. Here, we investigate if and how these representation schemes are affected by age-related hearing loss in the gerbil.

The quiet-aged gerbil is an attractive model for studying the pathology of age-related deficits in speech-in-noise perception and has often been used to study age-related hearing loss. Therefore, much is known about its age-related cochlear and central auditory pathologies (e.g., Schmiedt, 2010; Radtke-Schuller et al., 2015; Kessler et al., 2020). Furthermore, the gerbil has good low-frequency hearing, similar to humans, and important to translate speech encoding and perception, for which spectral energy at low frequencies is fundamental (Ryan, 1976; DePaolis et al., 1996). In addition, gerbils can easily be trained to indicate their behavioral discrimination between different logatomes (Sinnott et al., 1997; Sinnott and Mosteller, 2001). Importantly, young, normal-hearing gerbils experience the same confusions between different vowels as humans, although they need higher SNRs for equivalent performance (Jüchter et al., 2022).

Our previous studies on behavioral vowel discrimination in noise by young-adult gerbils revealed which vowels were difficult and which were easy to discriminate from each other (Jüchter et al., 2022). Furthermore, neural vowel discrimination at the level of a single AN fiber agreed with behavioral discrimination when applying a spike timing-based discrimination metric (Heeringa and Köppl, 2022). Here, our aim was to study single auditory nerve fiber and behavioral vowel discrimination in quiet-aged gerbils, to elucidate some of the underlying mechanisms of the age-related deficit in speech-in-noise perception. We showed that mild age-related hearing loss provided an advantage in terms of vowel discrimination at the level of AN spiking patterns, while behavioral discrimination abilities for vowels in noise were not significantly affected in quiet-aged gerbils.

## 2. Materials and methods

### 2.1. Animals

For the neurophysiological part of this study, seven young-adult (three female) and five quiet-aged (one female) Mongolian gerbils (*Meriones unguiculatus*) were used. Animals weighed between 63 and 120 grams, were of either sex, and were free of middle-ear infections. Young-adult gerbils were aged 3.5–6.0 months and quiet-aged gerbils were aged 36.7–41.4 months. Hearing thresholds were determined by measuring auditory brainstem responses (ABRs). Only animals with an ABR threshold to chirps <60 dB sound pressure level (SPL) were included, thereby excluding several quiet-aged gerbils with severe age-related hearing loss. This criterion was necessary to make sure all animals could process the speech stimuli, which had a fixed stimulus SPL (see below). Data of six out of seven young-adult gerbils were published in a previous report (Heeringa and Köppl, 2022).

For the behavioral experiments, a different group of nine young-adult (four female) and ten quiet-aged gerbils (three female) was used, with weights between 60 and 87 grams. The age range for the young-adult gerbils was 4–9 months and for the quiet-aged gerbils 33–43 months, at the time of data collection. Binaural ABR thresholds to clicks were < 52 dB peak equivalent SPL (dB pe SPL) for the young-adult gerbils and < 69 dB pe SPL for the quiet-aged gerbils, measured before the training started or during the behavioral testing. Behavioral data of four out of nine young-adult gerbils were published previously (Jüchter et al., 2022).

All animals were born at the University of Oldenburg animal house and were group housed in a controlled, quiet environment (average sound levels L_Aeq_ of 48 and 55 dB SPL outside and during working hours, respectively) to minimize noise-induced hearing loss. All experimental procedures were reviewed and approved by the ethics authorities of Lower Saxony, Germany, under permit numbers AZ 33.19-42502-04-15/1990, AZ 33.19-42502-04-21/3695, and AZ 33.19-42502-04-21/3821.

### 2.2. The auditory brainstem response (ABR)

For the neurophysiological experiments, ABRs were used to determine hearing thresholds before recording from single-unit AN fibers and to monitor cochlear health during the ongoing experiment. ABRs were recorded during the presentation of chirps (0.3–19 kHz, 4.2 ms duration). Stimulus levels were typically separated by 5 dB and were presented randomly (200–400 repetitions per level). Acoustic stimuli were generated and calibrated using custom-written software in MATLAB (MathWorks) and an external audio card (Hammerfall DSP Multiface II, RME Audio; 48 kHz sampling rate). After preamplification (HB7, TDT), stimuli were presented through a small speaker (IE 800, Sennheiser) that was sealed into the ear bar. After each (re)placement of the ear bar, a calibration file was obtained by measuring SPL near the eardrum with a miniature microphone (ER7-C, Etymotic Research) sealed in the same ear bar, amplified by a microphone amplifier (MA3, TDT). To record ABRs, one platinum needle electrode was placed subcutaneously ventral of the ear canal, and one was placed in the ipsilateral neck muscle. ABRs were amplified (10,000 times) and bandpass filtered (0.3–3 kHz) using an amplifier (ISO 80, World Precision Instruments), and recorded using the same Hammerfall audio card (48 kHz sampling rate) controlled by custom-written software (MATLAB). ABR thresholds were defined as the lowest sound level at which clear ABR waves were still visually distinguishable, and at which wave I had an amplitude of 4 μV or higher.

For the behavioral experiments, ABRs were measured once to make sure the animal was able to hear the stimuli needed for training. Recordings of the ABRs were obtained similarly as described above, with a few exceptions. ABRs were recorded during the presentation of clicks (0.2–15 kHz, 40 μs duration) with 10-dB level steps. No microphone amplifier was used. Furthermore, the stainless-steel needle recording and reference electrodes were placed subcutaneously at the vertex and in the neck on the midline, respectively. Finally, ABR thresholds were defined using custom MATLAB software written by one of the authors (R.B.) using the procedure described in Suthakar and Liberman (2019), which was visually cross-checked for each threshold. All animals had click ABR thresholds <70 dB pe SPL and proceeded to the behavioral training.

### 2.3. Single-unit auditory nerve fiber recordings

#### 2.3.1. Surgical procedures

Gerbils were anesthetized with 135 mg/kg ketamine (Ketamidor, WDT) and 6 mg/kg xylazine (Xylazin, Serumwerk) diluted in saline (0.9% NaCl) and injected intraperitoneally. Anesthetic depth was maintained by supplementary subcutaneous ketamine/xylazine injections. One-third of the initial dose was injected hourly, and one-sixth of the initial dose was provided upon a positive hind paw reflex. Additionally, three out of five quiet-aged gerbils received a preemptive injection of the antiphlogistic agent meloxicam (Metacam, Boehringer Ingelheim; 1 mg/kg). Two out of five quiet-aged gerbils were tracheotomized but breathed unaided. Additional oxygen was provided (1.5 L/min) in front of the mouth or tracheotomy tube throughout the experiment to all animals. Heartbeat, heart rate, muscle potentials, and breathing rate were constantly monitored through electrocardiogram recordings using intramuscular needle electrodes in the front and contralateral hind leg. Body temperature was maintained at 38°C using a rectal probe and a homeothermic blanket (Harvard Apparatus).

The skull of the animal was fixed in a bite bar (Kopf Instruments), with the head mount glued to the exposed frontal skull using dental cement. The right bony ear canal was exposed by removing the pinna. The ear bar, which contained the speaker (IE 800, Sennheiser) and miniature microphone (ER7-C, Etymotic Research), was sealed to the ear canal using petroleum jelly. A small opening was made in the dorsal-lateral bulla to prevent negative pressure buildup in the middle ear.

The auditory nerve was visualized using a dorsal approach as follows. The bony structures overlying the right cerebellum, including parts of the occipital, parietal, and temporal bone, were removed carefully. After a duratomy, the cerebellum was partially aspirated until the brainstem overlying the AN was exposed. The AN was visualized by placing one or two small balls of paper tissue (< 0.5 mm diameter), drenched in saline, between the brainstem and the temporal bone.

#### 2.3.2. Single-unit recordings

Glass micropipette electrodes (BF120F-10, Science products) were pulled on a P-2000 puller (Sutter Instruments) and filled with 3 M KCl. Impedances were typically between 20 and 40 MΩ. Electrodes were mounted into an electrode holder that was attached to an inchworm motor controller (6000 ULN, Burleigh), which was held by a micromanipulator (Märzhäuser). Under visual control and using the micromanipulator, electrodes were placed just above the AN fiber bundle. Electrodes were advanced through the AN bundle in small steps (1–5 μm; 6005 ULN handset, Burleigh). Meanwhile, a broadband-noise search stimulus (50–70 dB SPL) was played through the speaker. Signals recorded from the glass electrode were amplified (WPI 767), filtered for line-frequency noise (50/60 Hz; Hum Bug, Quest Scientific), made audible through a speaker (MS2, TDT), visualized on an oscilloscope (SDS 1102CNL, SIGLENT Technologies), and digitized (RX6, TDT; 48,828 Hz sampling rate) before being displayed on a personal computer using custom-written MATLAB software.

After a single unit was isolated, a quick audiovisual estimate of the fiber’s best frequency (BF) and threshold was obtained. Tone bursts at around 10 dB above the estimated threshold (50 ms ON – 190 ms OFF time, 5 ms cosine rise/fall times, 5 repetitions, 10 to 20 linear frequency steps spanning approximately 1.5 octave) were presented to determine the unit’s BF from a frequency-response curve. Subsequently, tone bursts at BF were presented at a range of levels (3 dB step size, 10 repetitions) to determine the unit’s threshold from a rate-level function. After recording responses to the consonant-vowel-consonant (CVC) stimuli, as described below, 24 s of neural activity in the absence of acoustic stimuli were recorded to determine the unit’s spontaneous rate (SR). When this recording was not available (*n* = 103/194 of units), SR was estimated from the randomly inserted silent trials of the rate-level-function recordings (total duration of 800 ms). Furthermore, clicks were presented (97 dB pe SPL, 5 ms delay, 20 ms acquisition time, condensation click, 300 repetitions) to determine response latency. In a small subset of units (*n* = 4 in young-adult and *n* = 4 in quiet-aged gerbils), responses to amplitude-modulated tones were collected (128 Hz modulation frequency, 100% modulation depth, carrier frequency at BF or one octave above BF, 2.5 s duration, 20 ms delay, 20 ms cos^2^ rise and fall time, levels ranging from 10–80 dB SPL, 10 repetitions per level). All stimuli, except for the clicks, were calibrated using custom-made MATLAB software.

#### 2.3.3. Consonant-vowel-consonant stimuli

CVCs derived from the Oldenburg Logatome speech corpus (OLLO) database (Meyer et al., 2010). Vowels were selected based on the outcomes of a behavioral study in young-adult gerbils of the same colony that had demonstrated common and uncommon confusions between all vowels available in the database (Jüchter et al., 2022). Three vowels were chosen from that study: two that were difficult to discriminate from each other (/eː/ vs. /iː/) and one that was easy to discriminate from the other two (/aː/). The outer consonant was fixed at /b/, resulting in the following spoken logatomes: ‘behb’, ‘bieb’, and ‘bahb’. Logatomes were spoken by a native German, female speaker (code S01F in the OLLO database), who had an average *f_0_* of 259 Hz across the three logatomes. Formant frequencies of these three vowels are listed in Table 1 and were determined as follows. Center phonemes of the logatome stimuli were cut out from the full signal at the AN response analysis window. These cutout signals were filtered with a single pole pre-emphasis filter, which flattens the overall spectral shape, that is, approximates a white noise more closely. This is a prerequisite for the subsequent formant extraction step, which is performed by linear prediction (MATLAB, lpc function, 16th order). The resulting filter pole center frequencies and bandwidths were chosen by a set of criteria: *f_2_* must be greater than *f_1_*, *f_1_*, and *f_2_* have to lie within the known general limits for *f_1_* and *f_2_*, respectively, of all vowels, and the bandwidths have to be within reasonable limits. *F_0_* as well as the drift of *f_0_* are also specified in Table 1. Average *f_0_* was extracted using the maximum of the autocorrelation function of the full cutout phoneme, and instantaneous *f_0_* was extracted using the (discrete) derivative of the instantaneous complex phase of the band-pass (50–400 Hz) filtered signal. The phase was calculated with the help of a Hilbert transform.

**Table 1:** Fundamental and formant frequencies of the presented vowels.

	Neurophysiology (S01F)				Behavior (frequency range of all speakers)		
	*f_0_* (Hz)	*f_1_* (Hz)	*f_2_* (Hz)	*f_0_* drift (Hz)	*f_0_* (Hz)	*f_1_* (Hz)	*f_2_* (Hz)
/aː/	257	850	1,275	255–260	105–258	566–850	1,122–1,352
/eː/	257	440	2,450	254–259	106–267	279–466	1,914–2,466
/iː/	262	275	2,600	258–267	109–277	219–316	2,076–2,575

Acquisition duration of one trial with a logatome was 1.15 s, logatome delay was 0.125 s, average length of the logatomes was 0.57 s, and each logatome was presented 60 times. Logatomes were presented at 65 dB SPL against a speech-shaped background noise (ICRA1) derived from the ICRA database (Dreschler et al., 2001) at 5 dB SNR. One excerpt from the ICRA1 noise (duration of 1.15 s) was used as frozen noise background for all stimulus presentations during single unit recordings. Thus, the background noise cannot be a factor explaining variability between responses. Furthermore, the putatively detrimental effects of background noise cannot be averaged out when the noise is frozen between repetitions. We found previously that having a different token of the ICRA1 background noise does not change spike timing-based vowel discrimination in AN fiber responses of young-adult gerbils (Heeringa and Köppl, 2022), suggesting that this particular frozen noise token did not strongly affect the outcomes of the analyses. Noise onset and offset at the beginning and end of the recording time were ramped (10-ms cos^2^ ramps).

#### 2.3.4. Single-unit characterization

All neural signals were bandpass filtered (300–3,000 Hz) and revisited offline for spike detection threshold on a trial-by-trial basis. To determine BF more accurately, rate-frequency curves were fitted using a smoothing spline function. BF was defined by the location of the peak of this fitted smoothing spline function along the frequency axis. Threshold was determined from the rate-level function, defined as the tone level evoking a firing rate larger than mean (SR) + 1.2*std (SR) and at least 15 spikes/s above the mean SR. SR was determined from the 24 s long recording in silence or from the randomly presented silent trials throughout the tone presentations for the rate-level function at BF, which had a total length of 800 ms. The SR was used to separate the data between low- and high-SR fibers, with the cut-off between these populations at 18 spikes/s (Schmiedt, 1989).

To confirm that the recorded unit was an AN fiber, and not accidentally recorded from the cochlear nucleus, several checks were carried out. Spike waveforms were checked for the absence of a prepotential, the response pattern to tones at 20–30 dB above threshold were checked for having a primary-like shape, and the shape of the rate-level function was required to fall into one of the three known shapes of AN rate-level function (straight, sloping saturating, or flat saturating; Winter et al., 1990; Heeringa et al., 2023). When responses to clicks were obtained, click latency was determined and matched to our own click latency vs. BF distribution. When any of these checks failed, the unit was excluded from further analysis. Furthermore, inter-spike intervals (ISIs) were assessed to confirm single-unit isolation. Units with ISIs <0.6 ms, that is the absolute refractory period of AN fibers (Heil et al., 2007), were excluded from further analysis since these spikes likely derived from more than one AN fiber.

#### 2.3.5. Analysis of vowel responses

We found previously that a spike timing-based discrimination metric, the Δ correlation index (ΔCI), agreed with behavioral discrimination abilities in young-adult gerbils (Heeringa and Köppl, 2022; Jüchter et al., 2022). Therefore, we also explored the effects of aging on this neural vowel discrimination metric. ΔCI is calculated as follows. First, the shuffled autocorrelogram (SAC) was calculated for each individual vowel response, according to Louage et al. (2004). The SAC differs from the autocorrelogram (or all-order inter-spike interval histogram) in that it is based on intervals between spikes across spike trains from different trials, rather than the intervals between spikes within one spike train. SACs were calculated using a bin width of 20.48 μs and were normalized for firing rate (*r*), repetitions (number of trials) (*N*), bin width (Δ*τ*), and analysis window duration (*D*) by dividing the resulting SAC with the following correction factor (*C_SAC_*):


$$C_{SAC}=N\left(N-1\right)\times r^{2}\times \Delta \tau \times D$$

Only spiking activity recorded during the steady-state part of the vowel was analyzed (analysis window duration *D* of 233 ms). Next, the crossed autocorrelogram (XAC) was calculated for each combination of vowel responses recorded in one AN fiber, that is /aː/ vs. /eː/, /aː/ vs. /iː/, and /eː/ vs. /iː/. The XAC can be described as the all-order interval histogram of spikes across responses from two different stimuli, rather than between responses to one stimulus as in the SAC (Louage et al., 2004). Since there are no identical repetitions, as in the SAC, and two different rates (*r_1_* and *r_2_*, referring to the rates of vowel 1 and vowel 2), the correction factor for the XAC (*C_XAC_*) is described as follows:


$$C_{XAC}=N^{2}\times r_{1}\times r_{2}\times \Delta \tau \times D$$

The same analysis window, start time, and stop time was applied to all three vowel responses. Since the background noise was frozen between all recordings, this also rules out background noise-based variability. The correlation index (CI), defined by the maximum peak height of the normalized SAC, was shown to be a measure of trial-by-trial temporal similarity between responses to the same stimulus (Joris et al., 2006) and has a strong correlation to the classical vector strength measure for phase locking (Kessler et al., 2021). Similarly, the CIx, defined by the maximum peak height of the normalized XAC, can be regarded as a measure of temporal similarity between spiking responses to two different stimuli, with low values meaning low similarity and thus suggesting good discrimination. To account for cases where temporal similarity of responses to the same vowel was already low, the difference between CI and CIx was taken as a spike timing-based discrimination metric, here termed ΔCI (Heeringa and Köppl, 2022). A high ΔCI value reflects temporal dissimilarity between neural responses to the two compared vowels, whereas a low ΔCI value reflects temporally similar responses to the two vowels, regardless of the overall temporal precision of spiking of a given AN fiber.

Neural vowel representation schemes were constructed based on vowel responses from AN fibers recorded in young-adult and quiet-aged gerbils according to methods described in detail in Heeringa and Köppl (2022). Specifically, the ALIR (averaged localized interval rate) and the dominant component scheme were constructed based on the fast-Fourier transform (FFT) of the all-order inter-spike interval histogram (ISIH; 31.25 ms length, 256 bins) (Sachs and Young, 1980; Delgutte and Kiang, 1984b). The all-order ISIHs were calculated over all trials with responses to one vowel during an analysis window when the vowel was present. For the ALIR, the all-order ISIHs were first converted to interval/s before calculating the FFT. Subsequently, the peak height of the ISIH FFT at each harmonic of *f_0_* was averaged for all units with a BF near that harmonic (Sachs and Young, 1980). The ALIR of each vowel response was plotted as a function of the *f_0_* harmonics for young-adult and quiet-aged gerbils. By excluding the correction for interval rate in the all-order ISIH and plotting it as a probability histogram, the FFT produces a purely temporal measure (Moissl and Meyer-Base, 2000). Since the unit of a first-order ISIH FFT is a temporal coding measure ranging from 0–1 (the synchronization index), the all-order ISIH FFT is expressed as the squared synchronization index (*SI^2^*). The dominant component scheme was obtained by plotting the frequency of the highest peak at or near *f_0_* or its harmonics in this temporal ISIH FFT as a function of the fiber’s BF, following methods described by Delgutte and Kiang (1984b).

To determine total temporal power at or away from the vowel’s formant frequencies, the all-order ISIHs expressed as ISI probability, and their FFT were considered, as described above. First, a general overview of the AN response was obtained by plotting the median all-order ISIH FFT for each vowel and each age group across all AN fibers with a low-BF and across those with a high-BF (cut-off at 3.5 kHz; Huet et al., 2016). Additionally, peaks in the ISIH FFT were further analyzed to determine synchronization (that is, temporal coding strength) at *f_0_*, at the harmonics closest to the vowel’s formant frequencies, and at all *f_0_* harmonics combined, according to methods described by Wong et al. (1998). At *f_0_* and its harmonics, the highest peak in the all-order ISIH FFT, expressed as the *SI^2^* value, in an *f_0_*-wide window was determined. Synchronization at the formant frequencies was calculated by adding the *SI^2^* at the harmonics closest to *f_1_* and *f_2_*,


$$\mathrm{Formant}\;\mathrm{synchronization}=\underset{k=L}{\sum }SI^{2}\left(k.f_{0}\right)$$

*L* indicates the list of harmonics which are closest to *f_1_* and *f_2_*: for /aː/ L = [3, 4, 5], for /eː/ L = [2, 9, 10, 11], and for /iː/ L = [2, 10]. Higher formant frequencies were often buried in the noise and synchronization to these formants was typically not observed in the neural responses. Furthermore, *f_1_* and *f_2_* have been shown to account for most variability in discriminating between vowels (Peterson and Barney, 1952; Jüchter et al., 2022). Therefore, only *f_1_* and *f_2_* were considered in the analysis of the formant synchronization. The total synchronizing power of the ISIH FFT was calculated as follows,


$$\mathrm{Total}\;\mathrm{synchronizing}\;\mathrm{power}=\overset{N}{\underset{k=1}{\sum }}SI^{2}\left(k.f_{0}\right)$$

where *N* indicates the total number of *f_0_* harmonics included in the analysis. Here, we used the first 15 harmonics of *f_0_*, relating to a maximum frequency of 4.16 kHz.

#### 2.3.6. Responses to amplitude-modulated tones

Vector strength (VS) to the modulation frequency of the amplitude-modulated tones (Goldberg and Brown, 1969) was calculated in the time window between 20 ms after the start of the stimulus until the end of the stimulus. VS represents the tendency of a unit to phase lock to the period of the modulation frequency, and was calculated by


$$VS=\frac{1}{n}\left|\overset{n}{\underset{j=1}{\sum }}e^{i\varphi \left(j\right)}\right|$$

where *n* represents the total number of spikes, *φ(j)* is the phase of the *j*-th spike relative to the period of the modulation frequency, and *i* is the complex number √(−1). Significance of the VS was determined by


$$p=e^{-n.VS^{2}}$$

When *p* < 0.001 and *n* > 50, VS was considered significant (Mardia and Jupp, 2000).

### 2.4. Behavioral vowel discriminability

#### 2.4.1. Behavioral paradigm

Gerbils were trained to detect a vowel change in a continuously repeating reference of CVC logatomes with a fixed outer consonant. When the vowel in the repeating reference logatome changed, the animal had to indicate the detection of the change by jumping off a pedestal. If this was correct, the animal was rewarded with a 10-mg food pellet. The order of reference and target vowel were randomized across sessions and between animals. For the behavioral part of the study, an extended set of CVCs including various central vowels and outer consonants were used, but only the behavioral responses to the CVCs used in the AN recordings are analyzed and reported here (72 trials per animal).

All CVCs derived from the OLLO speech material database and were presented against continuous ICRA1 background noise at the same SNR (+5 dB) and SPL (65 dB) as the ones used in AN recordings. CVCs used in the behavioral tests were spoken by two male and two female speakers, including the female speaker who spoke the CVCs used in the AN recordings, and included two utterances per speaker. Each consecutive CVC was randomly chosen from these speakers and utterances. Hence, only a change in vowel, not speaker identity, needed to be reported. For the discrimination of, for example, /aː/ vs. /eː/, both an /aː/ deviant in a stream of repeating /eː/, and an /eː/ deviant in a stream of repeating /aː/ is considered. Table 1 shows the frequency ranges of the fundamental and formant frequencies of the three vowels spoken by the speakers listed above. *F_0_* and formant frequencies were derived as described above (at *2.3.3. Consonant-vowel-consonant stimuli*) except that center phonemes of the logatome stimuli were cut out from the full signal at the phoneme label boundaries provided with the OLLO speech corpus. For a detailed description of the behavioral setup and procedure, see Jüchter et al. (2022).

#### 2.4.2. Analysis of behavioral data

Response latencies and detection probabilities were measured to quantify the discriminability of the vowels. For misses and correct rejections in catch trials, a maximum response time of 1.5 s was used. Similarity matrices filled with ranks that were based on the response times of the gerbils for the discrimination between the different CVCs were entered into the multidimensional scaling (MDS) procedure PROXSCAL (Busing et al., 1997) in SPSS (IBM; version 27) for generating perceptual maps. MDS translates the differences in response time ranks to distances in a multidimensional space representing the perceived stimulus similarity by spatial proximity. Long response times are reflected by short distances in the perceptual map, and thus represent poor behavioral discriminability between two vowels. Short response times are shown as large distances in the perceptual map and thus indicate good behavioral discriminability between the two vowels. Values for the sensitivity-index d-prime (d’) were calculated, applying the inverse cumulative standard normal distribution function Φ^−1^ to the hit (H) and false-alarm rates (FA): d’ = Φ^−1^ (H) – Φ^−1^ (FA) (Macmillan and Creelman, 2004). For more details about the behavioral data analyses, see Jüchter et al. (2022).

### 2.5. Statistics

Each data distribution that was entered into statistical analyses was tested for normality using the Shapiro–Wilk test. When composite normality was a reasonable assumption (*p* > 0.05 on the Shapiro–Wilk test), parametric tests were used to evaluate group differences. Specifically, to evaluate effects of age group and vowel comparison on response latencies and d’-values from the behavioral experiments, two-way ANOVAs followed by post-hoc two-sample *T*-tests were used. For ABR thresholds, a normal distribution could also be reasonably assumed and a two-sample *T*-test was used to determine significant differences between age groups. For the other data distributions, normality could not be assumed, and one of the following non-parametric tests were used as appropriate: Mann–Whitney *U* tests, Friedman tests, or Wilcoxon Signed Rank tests. Bonferroni corrections were used to correct for multiple comparisons. Statistical analyses were carried out in SPSS (IBM; version 27) and MATLAB (MathWorks; version R2023b) using the Statistics and Machine Learning Toolbox™ (version 23.2).

## 3. Results

### 3.1. Behavioral vowel discrimination did not decline in gerbils with age-related hearing loss

**Figure 1:**
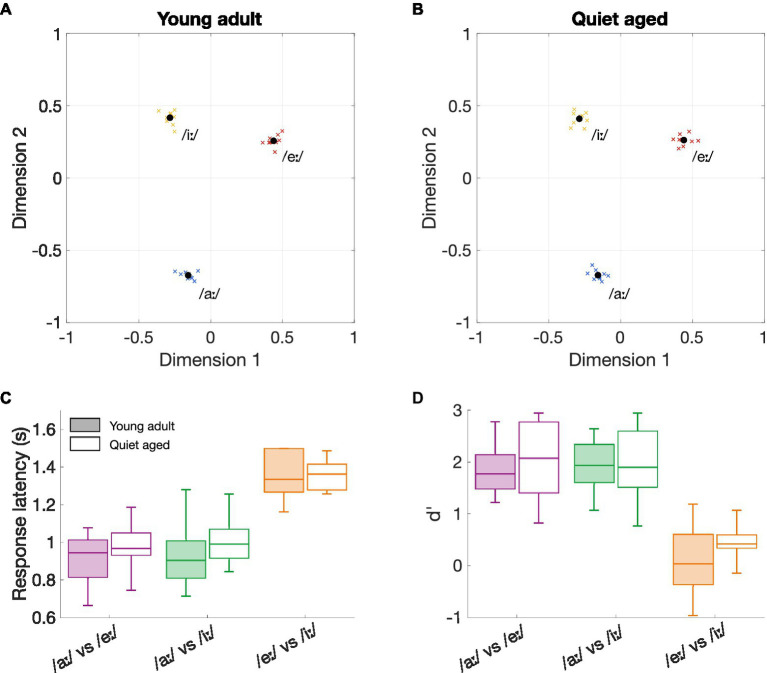
The effects of aging on behavioral vowel discrimination. **(A)** Perceptual map of young-adult gerbils (*n* = 9). Vowels in close proximity to each other indicate a more difficult discriminability. The perceptual map was constructed based on the three logatomes that were used in the neurophysiological study, with vowels /aː/, /eː/, and /iː/ at 5 dB SNR. Blue, red, and yellow crosses represent data from individual gerbils for the vowels /aː/, /eː/, and /iː/, respectively; black circles represent the group mean. **(B)** Perceptual map of quiet-aged gerbils (*n* = 10). **(C)** Boxplots of the response latencies for the three vowel discriminations of young-adult (filled box plots) and quiet-aged gerbils (open box plots). The boxes indicate the 25th and 75th percentiles and the median. The whiskers indicate the upper and lower limits of the range of data points. **(D)** Boxplots of the sensitivity index d’ for the three vowel discriminations of young-adult (filled box plots) and quiet-aged gerbils (open box plots).

Figure 1 shows the perceptual maps of young-adult (Figure 1A) and quiet-aged gerbils (Figure 1B), for the vowels that were included in the neurophysiological part of this study. These maps visualize which vowel discrimination is easy and which difficult. Proximity between the three vowels as well as their locations on the perceptual map are remarkably similar between the two age groups. For all animals, the vowels /eː/ and /iː/ were more difficult to discriminate (closer to each other on the perceptual map) than /iː/ vs. /aː/ or /eː/ vs. /aː/.

To investigate these putative perceptual similarities between young-adult and quiet-aged gerbils further, response latencies and the sensitivity index d’ were explored in more detail. The results indicated a significant main effect for vowel comparison on response latency (two-way ANOVA, factor vowel comparison: *F*(2, 51) = 59.31, *p* = 4.92*10^−14^; Figure 1C). As expected from the perceptual maps, the discrimination /eː/ vs. /iː/ resulted in longer response latencies than the discriminations /aː/ vs. /eː/ (M_Diff_ = 0.41, 95%-CI[0.30, 0.51], *p* = 1.50*10^−12^) and /aː/ vs. /iː/ (M_Diff_ = 0.39, 95%-CI[0.29, 0.50], *p* = 6.13*10^−12^) when data of both young-adult and quiet-aged gerbils was combined. There was no significant effect of age group on response latency and no factorial interaction (factor age group: *F*(1, 51) = 1.66, *p* = 0.20; age group × vowel comparison: *F*(2, 51) = 0.32, *p* = 0.73). Values for d’ were also found to be significantly different for the different vowel comparisons (two-way ANOVA, factor vowel comparison: *F*(2, 51) = 48.49, *p* = 1.60*10^−12^; Figure 1D). Specifically, Bonferroni corrected post-hoc testing revealed that the d’-values for the /eː/ vs. /iː/ discrimination were significantly lower compared to the d’-values for the /aː/ vs. /eː/ (M_Diff_ = 1.68, 95%-CI[1.19, 2.17], *p* = 6.46*10^−11^) and /aː/ vs. /iː/ (M_Diff_ = 1.67, 95%-CI[1.18, 2.16], *p* = 7.84*10^−11^). The two-way ANOVA revealed no significant effect of age on d’-values and no significant factorial interaction (factor age group: *F*(1, 51) = 1.20, *p* = 0.28; age group x vowel comparison: *F*(2, 51) = 0.37, *p* = 0.69). Together, these results indicate that behavioral discrimination of vowels in background noise was highly determined by vowel comparison, rendering age as an unimportant factor.

### 3.2. Neural vowel discrimination was enhanced in single auditory-nerve fibers of quiet-aged gerbils

**Figure 2:**
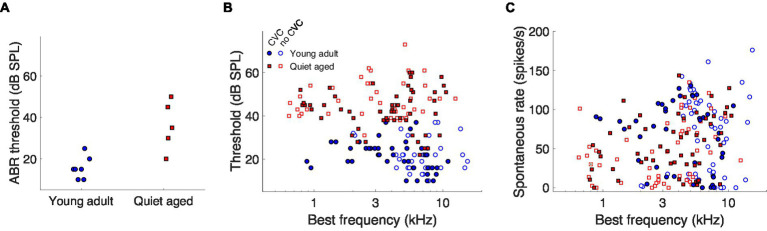
The effects of aging on neurophysiological thresholds and auditory-nerve spontaneous rates. **(A)** ABR thresholds to chirps of the young-adult (*n* = 7) and quiet-aged (*n* = 5) gerbils from which single-unit recordings were collected. **(B)** Single-unit AN fiber thresholds for tone bursts at BF as a function of the fiber’s BF. Data of young-adult and quiet-aged gerbils are plotted as blue circles and red squares, respectively. Units in which responses to at least one vowel were also recorded are represented by the filled symbols. **(C)** Single-unit AN fiber SRs as a function the fiber’s BF. Symbols represent the same as in panel **(B)**.

The quiet-aged gerbils, from which AN fiber responses to the vowels were recorded, had a mild to moderate degree of age-related hearing loss. ABR thresholds to chirps were significantly elevated in the quiet-aged compared to the young-adult gerbils by 20 dB on average (two-sample *T*-test: *T*(10) = 4.02, *p* = 2.4*10^−3^; Figure 2A). BF and thresholds of all single AN units that were recorded from these animals are shown in Figure 2B. Fibers from which also CVC responses were recorded are indicated separately as filled symbols. BFs of fibers from which vowel responses were recorded, ranged from 880–11,000 Hz for young adults and from 785–10,600 Hz for quiet-aged gerbils. Note that the BFs of these fibers do not extend into the range of *f_1_* of the vowels /eː/ (440 Hz) and /iː/ (275 Hz) and barely encompass *f_1_* of /aː/ (850 Hz). Thresholds of AN fibers recorded in the quiet-aged gerbils were also significantly elevated compared to those of young-adult gerbils (Mann–Whitney *U*-test: *U* = 10.88, *p* = 1.38*10^−27^), with a median age-related elevation of 22 dB. Furthermore, the SR of fibers recorded in quiet-aged gerbils was significantly lower than those of young adults (*U* = 2.50, *p* = 0.013), consistent with what we have observed previously in a different sample of quiet-aged gerbils (Heeringa et al., 2020, 2023). Table 2 shows for each vowel and age group the number of AN fibers for which responses to CVCs were recorded.

**Table 2:** Number of AN fibers recorded for each vowel and age group.

	Young-adult gerbils	Quiet-aged gerbils
/aː/	*n* = 41	*n* = 54
/eː/	*n* = 35	*n* = 53
/iː/	*n* = 26	*n* = 49

**Figure 3:**
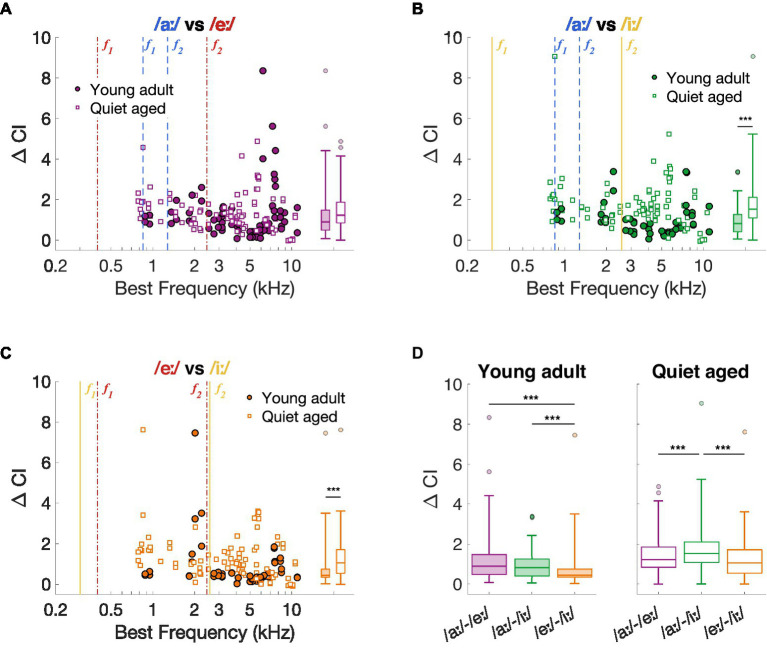
Neural spike-timing based vowel discrimination in young-adult and quiet-aged gerbils. **(A–C)** ΔCI for the vowel comparisons /aː/ vs. /eː/, /aː/ vs. /iː/, and /eː/ vs. /iː/ plotted as a function of the fiber’s BF [panel **(A–C)**, respectively]. Formant frequencies of the vowel /aː/ are shown in blue dashed lines, of /eː/ in red dotted-dashed lines, and of /iː/ in yellow solid lines (colors correspond to the plot titles). Significant differences between ΔCI from fibers of young-adult and quiet-aged gerbils are indicated by the horizontal bars between the boxplots shown at the right margin of each scatter plot. **(D)** ΔCI for the three vowel discriminations in young-adult and quiet-aged gerbils separately. Boxplots show the median, the 25th and 75th percentiles, the range of data points (without outliers), and outliers of the ΔCI values. Significant differences between vowel comparisons within each age group are indicated here. *** indicates *p* < 0.001.

As previously described, the spike timing-based discrimination metric ΔCI agreed with behavioral discrimination abilities in young-adult gerbils (Heeringa and Köppl, 2022; Jüchter et al., 2022). Therefore, we started with exploring the effects of aging on this neural vowel discrimination metric and compared the outcomes to the behavioral vowel discrimination abilities in quiet-aged gerbils. Figures 3A–C shows ΔCI as a function of BF for the vowel comparisons recorded in young-adult and quiet-aged gerbils. Across most of the BF range, ΔCI was higher in fibers of quiet-aged gerbils (open symbols) compared to young adults (closed symbols). Note that the fibers with a BF higher than the formants also temporally encode the formant frequencies of the vowels (see Section *3.4 “Temporal locking to fundamental and formant frequencies was stronger in aged fibers”*). Phase locking to these lower frequencies are enabled through the tail regions of the fiber’s tuning curve, which were likely reflected in the ΔCI of these high-BF fibers. Statistical testing showed that the median ΔCI was significantly higher in fibers of quiet-aged gerbils for the /aː/ vs. /iː/ and /eː/ vs. /iː/ comparisons (/aː/ vs. /eː/, *U* = 2.35, *p* = 0.057; /aː/ vs. /iː/, *U* = 4.88, *p* = 3.16*10^−6^; /eː/ vs. /iː/, *U* = 3.86, *p* = 3.48*10^−4^, Bonferroni corrected; Figures 3A–C, respectively).

The ΔCI data were replotted separately for each age group in Figure 3D to assess significant effects between stimulus comparisons. As shown in our previous study (Heeringa and Köppl, 2022), ΔCI differs significantly between the stimulus comparisons in fibers of young-adult gerbils (Friedman test: *χ*^2^ (2, 143) = 25.13, *p* = 3.50*10^−6^). Specifically, /eː/ vs. /iː/ revealed significantly lower ΔCI values compared to the two other comparisons (post-hoc Wilcoxon Signed Rank tests: /aː/−/eː/ vs. /aː/−/iː/, *Z* = 1.51, *p* = 0.39; /aː/−/eː/ vs. /eː/−/iː/, *Z* = 3.65, *p* = 7.83*10^−4^; /aː/−/iː/ vs. /eː/−/iː/, *Z* = 4.57, *p* = 1.43*10^−5^, Bonferroni corrected). These neural vowel discrimination results agree with behavioral discrimination abilities of young-adult gerbils using the same stimuli, where /eː/ vs. /iː/ is more difficult to discriminate from each other than /aː/ vs. /eː/ and /aː/ vs. /iː/ (Figure 1A) (see also Jüchter et al., 2022). In AN fibers of quiet-aged gerbils, ΔCI also differed significantly between stimulus comparisons (Friedman test: *χ*^2^ (2, 251) = 30.50, *p* = 2.38*10^−7^). However, in quiet-aged gerbils, /aː/ vs. /iː/ yielded significantly higher ΔCI values compared to the other stimulus comparisons (post-hoc Wilcoxon Signed Rank tests: /aː/−/eː/ vs. /aː/−/iː/, *Z* = 4.47, *p* = 2.31*10^−5^; /aː/−/eː/ vs. /eː/−/iː/, *Z* = 1.50, *p* = 0.40; /aː/−/iː/ vs. /eː/−/iː/, *Z* = 5.69, *p* = 3.80*10^−8^, Bonferroni corrected; Figure 3B). This does not agree with behavioral discrimination abilities of quiet-aged gerbils, who showed nearly identical discrimination performance as the young-adult gerbils (Figure 1B).

### 3.3. Qualitative temporal representation of formant frequencies was unaffected by aging

**Figure 4:**
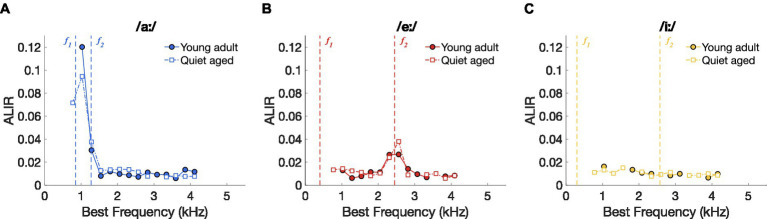
Vowel representation schemes of AN fibers. **(A)** ALIR scheme for responses to the vowel /aː/. Young-adult and quiet-aged gerbils are indicated by filled circles and open squares, respectively. Formant frequencies *f_1_* and *f_2_* are indicated by vertical dashed lines. **(B)** ALIR scheme for responses to the vowel /eː/. **(C)** ALIR scheme for responses to the vowel /iː/.

To further investigate the underlying mechanisms of the putatively improved vowel discrimination in the AN fibers of quiet-aged gerbils, spike timing-based representation schemes were constructed for the three separate vowels. Figures 4A–C shows the ALIRs of young-adult and quiet-aged gerbils’ AN fibers in response to the vowels /aː/, /eː/, and /iː/, respectively, which represent the average synchronized firing rate for fibers tuned to the *f_0_* harmonic nearest their own BF. ALIRs of fibers recorded in quiet-aged gerbils appeared very similar to ALIRs of young-adult gerbils, for all tested vowels. Peaks at formant frequencies for the vowels /aː/ and /eː/ were preserved with aging (Figures 4A,B) and the ALIR of the vowel /iː/ did not show peaks at formant frequencies for either age group (Figure 4C). Dominant component schemes, as described in Heeringa and Köppl (2022), were also examined, and revealed the same qualitative outcomes as described above for the ALIR (see Supplementary Figure S1).

Together, these data suggest that better temporal discrimination, as expressed as higher ΔCI values, in quiet-aged gerbils (Figure 3) does not correspond to qualitatively better temporal representation, when possible, e.g., in the ALIR of the vowel /iː/. Interestingly, ΔCI values were considerably higher in quiet-aged compared to young-adult gerbils for fibers tuned to BFs above 3.5 kHz (Figures 3A–C), the upper frequency limit for phase-locking in gerbils (Versteegh et al., 2011). Such high frequency fibers likely temporally code for the lower harmonics of the vowel’s power spectrum. The following section will explore this observation in more detail.

### 3.4. Temporal locking to fundamental and formant frequencies was stronger in aged fibers

**Figure 5:**
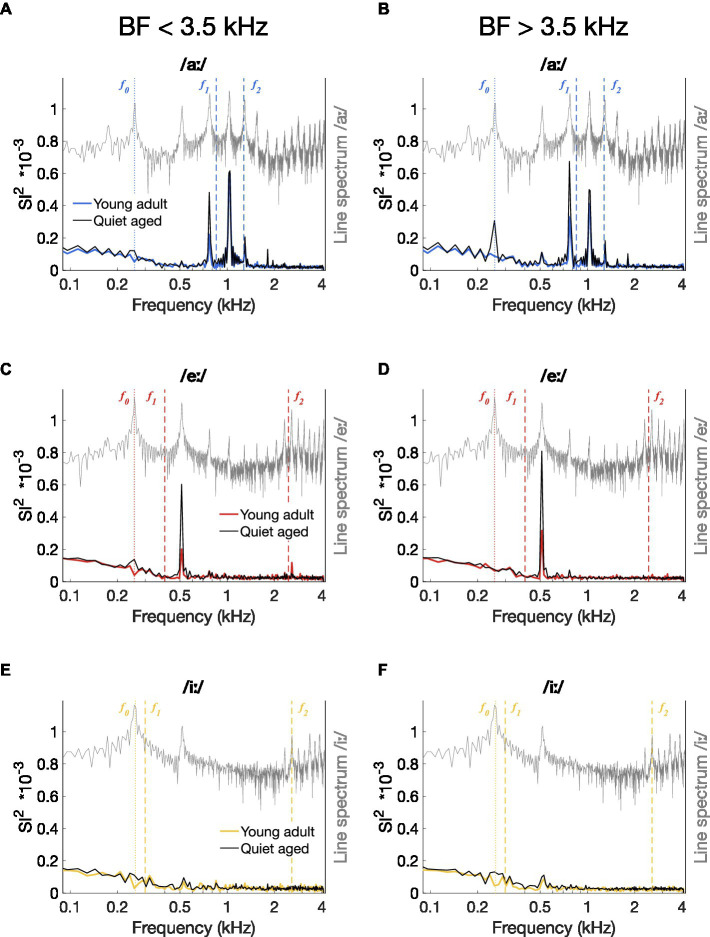
Median power spectrum of all-order inter-spike interval histograms. **(A–F)** Median ISIH FFT plots for responses to /aː/ [panels **(A,D)**], /eː/ [panels **(B,E)**], and /iː/ [panels **(C,F)**]. Median spectra were calculated separately for fibers with a BF < 3.5 kHz [panels **(A–C)**] and for fibers with a BF > 3.5 kHz [panels **(D–F)**]. Data of young-adult gerbils are represented by colored spectra; data of quiet-aged gerbils in black lines. Line spectra of the presented vowels are plotted in grey. Note that the spectrum of the background noise was not plotted to retain visibility of formant frequencies otherwise buried in the noise. Formant frequencies of the vowels are indicated by dashed vertical lines and *f_0_* is indicated as a dotted line.

An indication of temporal coding strength at different frequencies in the vowel responses was obtained by plotting the median ISIH FFTs of all AN fiber recordings. Figures 5A–C shows the median ISIH FFT of the low-BF fibers (BF < 3.5 kHz) and confirms stronger temporal coding in fibers of quiet-aged gerbils to the *f_0_* harmonic near *f_1_* for the vowels /aː/ and /eː/. High-BF fibers (BF > 3.5 kHz) also revealed enhanced temporal coding to the /aː/ and /eː/ vowels in quiet-aged gerbils, in addition to a peak at *f_0_* for vowel /aː/ which was absent in the data of the young-adult gerbils (Figures 5D–E). Median ISIH FFT plots do not show clear peaks near fundamental or formant frequencies in response to the vowel /iː/ for both young-adult and quiet-aged gerbils, in both BF groups (Figures 5C,F).

**Figure 6:**
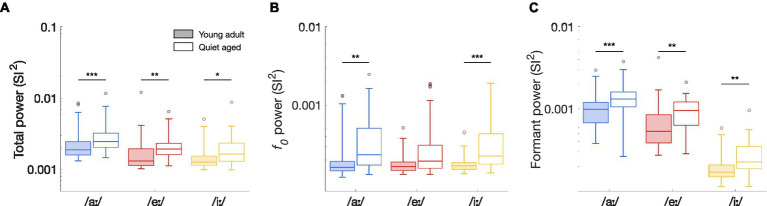
The effects of aging on ISIH FFT power. **(A)** Total synchronization power of the ISIH FFT of fibers in response to the vowels /aː/, /eː/, and /iː/, recorded from young-adult and quiet-aged gerbils depicted in filled and open boxplots, respectively. Legend applies to all panels. **(B)** Peak height at *f_0_*, derived from the ISIH FFT, in young-adult and quiet-aged AN fibers, in response to the three tested vowels. **(C)** Power at *f_0_* harmonics near the two formant frequencies, derived from the ISIH FFT, in young-adult and quiet-aged AN fibers. Boxplots show the median, the 25th and 75th percentiles, the range of data points (without outliers), and outliers, for AN fibers recorded from the young-adult and quiet-aged gerbils. Significant differences between age groups are indicated by horizontal bars. * indicates *p* < 0.05, ** indicates *p* < 0.01, *** indicates *p* < 0.001 in all panels.

To determine whether these differences in median ISIH FFT curves were significant, peak heights of the ISIH FFT at harmonic frequencies were compared between young-adult and quiet-aged gerbils. Total synchronization ISIH FFT power, as defined by the sum of the SI^2^-values at *f_0_* and the following 15 harmonics, was significantly increased with age for all three vowels tested (Mann Whitney-*U* tests: /aː/, *U* = 3.61, *p* = 9.17*10^−4^; /eː/, *U* = 3.45, *p* = 0.0017; /iː/, *U* = 2.92, *p* = 0.010 Bonferroni corrected; Figure 6A). To determine whether these increases derived from enhanced synchronization to *f_0_*, to formant frequencies, or both, the power in these frequency bands was studied separately. When only the peak at *f_0_* was considered, quiet-aged gerbils showed enhanced temporal coding in the ISIH FFT for responses to /aː/ and /iː/, but not to /eː/ (/aː/, *U* = 3.47, *p* = 0.0016; /eː/, *U* = 2.35, *p* = 0.056; /iː/, *U* = 3.89, *p* = 3.00*10^−4^, Bonferroni corrected; Figure 6B). Furthermore, when only peaks at harmonics near the formant frequencies were considered, the age-related increase in formant power was apparent for all three vowels (/aː/, *U* = 3.77, *p* = 4.93*10^−4^; /eː/, *U* = 3.36, *p* = 0.0023; /iː/, *U* = 3.32, *p* = 0.0027, Bonferroni corrected; Figure 6C).

### 3.5. Enhanced temporal coding in quiet-aged gerbils could be explained by stimulating closer to threshold

**Figure 7:**
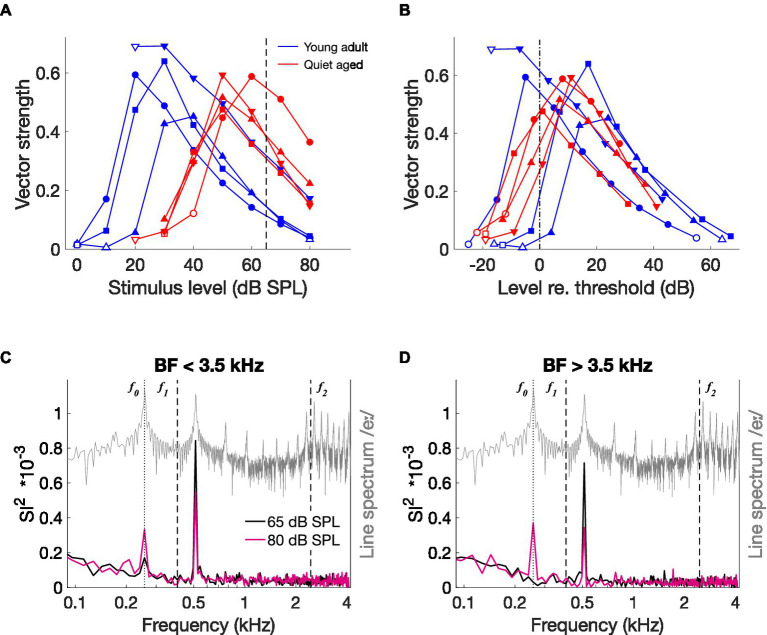
The effects of stimulus level on temporal coding. **(A)** Vector strength to the modulation frequency (*f_m_* = 128 Hz) of AM stimuli, as a function of different stimulus levels in four fibers of a young-adult and four fibers of a quiet-aged gerbil, indicated in blue and red traces, respectively. Open symbols indicate vector strength values that were not statistically significant (see Methods). The dashed line indicates the level of the CVC stimuli (65 dB SPL). **(B)** The same data as in panel **(A)**, replotted relative to the individual fiber’s threshold, indicated by the dashed-dotted line. Legend of panel **(A)** applies. **(C)** Median ISIH FFT of low-BF fibers from an old gerbil responding to /eː/ in noise (5 dB SNR) at 65 dB SPL (black trace) and at 80 dB SPL (pink trace) (*n* = 2 AN fibers). The line spectrum of /eː/ is plotted in grey. Note that the spectrum of the background noise was not plotted to retain visibility of formant frequencies otherwise buried in the noise. *f_0_*, *f_1_*, and *f_2_* are indicated in the plot. **(D)** Median ISIH FFT of three high-BF fibers from the same old gerbil, in response to the same stimuli as in panel **(C)**. Format is similar to panel **(C)**.

CVC stimuli were presented at the same level – 65 dB SPL – for young-adult and quiet-aged gerbils. However, quiet-aged gerbils had significantly higher thresholds (Figure 2B), meaning that the stimulus was presented closer to threshold in old compared to young-adult gerbils. This is important because temporal locking to low-frequency amplitude modulations is known to vary with stimulus level in AN fibers of normal-hearing cats, with a maximum at levels just above threshold (Joris and Yin, 1992). To determine if this relation is similar in young-adult and quiet-aged gerbils, we presented sinusoidal amplitude-modulated tones at BF at a range of different levels to a subsample of fibers recorded in young-adult and quiet-aged gerbils. Vector strength, a measure of temporal locking to periodic sinusoidal stimuli, steeply increased with increasing level and then decreased again less steeply, in fibers from both age groups (Figure 7A). At 65 dB SPL, indicated by the vertical dashed line in Figure 7A, vector strength in fibers of quiet-aged gerbils was higher than in those of young-adult gerbils. This can be explained by the difference in threshold sensitivity between young-adult and quiet-aged gerbils. The peak in the vector strength vs. stimulus level curve was within −5 and +20 dB of the fibers’ individual rate threshold, both for young-adult and quiet-aged gerbils (Figure 7B). Hence, the elevated thresholds in quiet-aged gerbils may partly explain enhanced temporal coding to vowels presented at the same SPL. If this is true, a ‘young adult-like’ vowel encoding in fibers of quiet-aged gerbils is predicted when the stimulus level of the vowel in noise is increased.

To test this hypothesis, we recorded responses to one vowel (/eː/) at two different levels from five single fibers of a quiet-aged gerbil, one at the regular 65 dB SPL and one at 80 dB SPL to compensate for the age-related threshold shift. Both levels included the background noise at 5 dB SNR relative to the SPL of the vowel. Like the ISIH FFT representation in Figure 5, responses of fibers with BF < 3.5 kHz are plotted separately from those with BF > 3.5 kHz. In the two low-BF fibers, the vowel in noise at 80 dB SPL resulted in a decrease of the peak at the first harmonic of *f_0_* (~ 550 Hz) and an emergence of a peak at the fundamental frequency (Figure 7C). This same pattern, but more pronounced, is seen in the high-BF fibers (Figure 7D). These data suggest that the enhanced temporal locking to the first harmonic in quiet-aged gerbils was due to stimulating closer to threshold. However, the stronger response to *f_0_* is not simply explained by a lower effective stimulation level. It appears to emerge specifically in old gerbils.

## 4. Discussion

Using behavioral experiments, we here showed that behavioral vowel discrimination was not significantly affected by aging. Response latencies and d’ sensitivity indices were similar between young adults and old gerbils. In contrast, temporal firing patterns were clearly dissimilar between young-adult and quiet-aged gerbils, for responses to all vowels. Contrary to intuition, a general improvement of temporal vowel encoding in noise across frequency was observed in old individuals. Furthermore, in quiet-aged gerbils, neural discrimination was not significantly worse for the behaviorally difficult /eː/ vs. /iː/ comparison, compared to the two easy comparisons. Representation schemes, based on the spectrum of the inter-spike interval histogram, revealed stronger encoding of both the fundamental and the first and second formant frequencies in AN fibers of quiet-aged gerbils. Elevated thresholds in combination with a fixed stimulus level can help explain these findings.

### 4.1. Why do AN fibers of quiet-aged gerbils display stronger temporal responses to vowels in noise?

Previously, our lab showed that temporal coding in AN fibers of quiet-aged gerbils was not affected by aging (Heeringa et al., 2020). In that study, temporal coding was evaluated from spiking activity in response to pure tones and white noise. Results showed that both encoding of the temporal fine structure, derived from tone-burst at BF and noise responses, and of the sound envelope, derived from noise responses, was unaffected by age-related hearing loss. Importantly, these results were obtained when comparing AN fiber responses between young adults and old gerbils at a fixed sensation level, i.e., every unit was stimulated at 20 dB above its individual rate threshold. In the current study, the stimulus had a fixed level of 65 dB SPL for both young-adult and quiet-aged gerbils, to enable a direct translation to the behavioral study. While temporal-fine structure encoding, as determined by vector strength to tone-bursts at BF, seems to be unaffected by aging for both fixed sound levels and fixed sensation levels (Heeringa et al., 2020), envelope encoding strongly varies as a function of level relative to the fiber’s threshold (Figure 7B). This can explain the higher synchronization indices especially to the lower frequencies (< 1 kHz) by fibers of quiet-aged gerbils.

Enhanced temporal coding by AN fibers has also been studied extensively following noise-induced cochlear damage. These studies revealed specifically that, even at equal sensation levels, noise-induced hearing loss causes a distorted tonotopy (e.g., Henry et al., 2016; reviewed in: Parida and Heinz, 2022a). This means that fibers respond more strongly to the low-frequency, off-BF part of stimuli, or even become hypersensitive to these tail frequencies. With noise-induced cochlear damage, frequency selectivity and tip-to-tail ratio are strongly correlated to distorted tonotopy. It is unlikely that a distorted tonotopy among AN fibers plays a major role in gerbils with age-related hearing loss, as the fiber’s frequency selectivity (measured 10 dB above of the most sensitive point) is not altered and tail hypersensitivity is absent in aged gerbils (Hellstrom and Schmiedt, 1996; Heeringa et al., 2020). Noise-induced deficits in frequency tuning and, consequently, distorted tonotopy are thought to be caused mainly by damage to outer hair cells (Liberman and Dodds, 1984; Parida and Heinz, 2022b), while quiet-aged gerbils, especially those with only mild-to-moderate hearing loss such as the ones involved in this study, have only minimal hair cell loss (Tarnowski et al., 1991). The main cause of age-related hearing loss in gerbils is strial dysfunction, resulting in a loss of the endocochlear potential (Schmiedt et al., 2002). Indeed, temporal coding deficits and associated tonotopic distortions were much less severe for experimentally induced metabolic hearing loss compared to a noise-induced hearing loss of the same degree (Henry et al., 2019). Nevertheless, a small degree of outer hair cell dysfunction and subsequent tonotopic distortion may have been present, as indicated by the enhanced *f_0_* response when presenting stimuli at the same sensation level (Figures 7C,D) as well as by a high proportion of low-frequency fine structure responses in high-BF fibers of old gerbils (see Figure 3D in Heeringa et al., 2020).

### 4.2. How is neural vowel encoding related to behavioral vowel discrimination?

Apart from the enhanced temporal coding, neural representation of naturally-spoken vowels in noise was qualitatively not affected by age-related hearing loss. This is similar to what has been found for vowel encoding by AN fibers following acoustic trauma (Geisler, 1989; Miller et al., 1997; Parida and Heinz, 2022b). In order to understand what is being said in adverse listening conditions, neural encoding of vowels only needs to be good enough for a perceptual discrimination from another vowel to remain possible. In our previous study, we have shown that the spike timing-based discrimination metric ΔCI agrees well with behavioral vowel discrimination abilities in young-adult gerbils (Heeringa and Köppl, 2022). Vowel combinations that were behaviorally difficult to discriminate from each other (/eː/ vs. /iː/) also showed lower values of ΔCI in responses of AN fibers. This suggests that, for young, normal-hearing gerbils, the limiting factor for behavioral discrimination of vowels may reside at or peripheral to the auditory nerve. Interestingly, quiet-aged gerbils displayed higher values of ΔCI in their AN fiber responses (Figure 3A). However, this neither translated to increased d’ values nor to shorter response latencies in old individuals. Furthermore, ΔCI of the different vowel comparisons did not comply with behavioral vowel discrimination abilities in quiet-aged gerbils. While the results suggest that ΔCI limits behavioral vowel discrimination in young-adult gerbils, this is not the case in quiet-aged gerbils. Rate-place coding can also contribute to neural vowel discrimination (Conley and Keilson, 1995). However, background noise strongly deteriorates the rate-place code in the auditory nerve, making it unlikely to be an additional limiting factor with age-related hearing loss (Sachs et al., 1983; Heeringa and Köppl, 2022). In addition, the ALIR, which contains an interval rate- and temporal-place coding, was not degraded with age-related hearing loss (see Figure 4). Therefore, these results suggest that additional age-related deteriorating processes in the central (auditory) system, starting at the synapse between the AN and its cochlear-nucleus targets, may affect vowel discrimination to the extent that the performance matches that of the young adults.

One such age-related deteriorating process to speech encoding in the central auditory system is known for the level of the auditory midbrain (inferior colliculus) (Khouri et al., 2011). Specifically, temporal coding of speech-like sounds and pulses are also enhanced in spiking activity of inferior colliculus neurons. However, temporal selectivity, that is to which modulation frequency the neuron responds best, as well as the heterogeneity in temporal responses between neurons decreases, which leads to a reduced benefit of pairing neurons for speech discrimination (Khouri et al., 2011; Parthasarathy et al., 2019). In other words, there is more redundancy in responses to speech among neurons of the inferior colliculus, resulting in putatively poorer speech encoding. An age-related decline in inhibition is thought to underlie these changes (Frisina and Walton, 2006; Caspary et al., 2008; Kessler et al., 2020).

### 4.3. What are the implications for humans with age-related hearing loss?

Age-related cochlear damage in the quiet-aged gerbil has been well characterized and proposed as a good model to make the translation to human age-related cochlear deficits (Schmiedt, 2010; Heeringa and Köppl, 2019). Similar to humans, quiet-aged gerbils in our colony also show substantial synapse loss, especially at the basal end of the cochlea, which processes the high-frequency sounds (Wu et al., 2020; Steenken et al., 2021). Among high-BF fibers, the low-SR fibers are particularly vulnerable to this synapse loss (Schmiedt et al., 1996; Heeringa et al., 2023). However, a reduction in the endocochlear potential, due to age-related damage to the stria vascularis, also affects the SR (Schmiedt, 1996; Wu et al., 2020). This can explain the general reduction of spontaneous rate, most strongly seen for the low-BF fibers (Figure 2C; Heeringa et al., 2020, 2023). Such cochlear changes in SR distribution, especially the loss of low-SR fibers, have been hypothesized to cause speech-in-noise perceptual deficits (Bharadwaj et al., 2014). However, as of yet, no strong evidence has been shown to support this hypothesis and much controversy remains (Ripley et al., 2022). The current study does not support the hypothesis that synapse loss causes a vowel-in-noise encoding or perceptual deficit.

The absence of an aging effect on vowel discrimination in gerbils seems to be in contrast to the common complaints of elderly humans about difficulties to understand speech in noisy conditions (Dubno et al., 1984; Fogerty et al., 2012; Füllgrabe et al., 2015). However, studies in humans that compared vowel vs. consonant perception showed that age-related problems with consonant perception are more common than those with vowels and also degrade speech intelligibility to a greater extent (Fogerty et al., 2012, 2015). By comparing behavioral with neural vowel discrimination using the same species and acoustic stimuli, we found here that although neural vowel encoding was altered by age, this did not significantly affect behavioral vowel encoding.

## Data availability statement

The datasets presented in this study can be found on Figshare: https://doi.org/10.6084/m9.figshare.23301377.v1.

## Ethics statement

The animal study was approved by Nds. Landesamt für Verbraucherschutz und Lebensmittelsicherheit (LAVES). The study was conducted in accordance with the local legislation and institutional requirements.

## Author contributions

CK, GK, and AH contributed to the conception and design of the study. AH and CJ collected and analyzed the data. RB provided software and assisted in the formal analysis. AH wrote the first draft of the manuscript. All authors contributed to manuscript revision, read, and approved the submitted version.

## References

[ref1] Bharadwaj H. M. Verhulst S. Shaheen L. Liberman M. C. Shinn-Cunningham B. G. (2014). Cochlear neuropathy and the coding of supra-threshold sound. Front. Syst. Neurosci. 8:26. doi: 10.3389/fnsys.2014.00026 24600357 PMC3930880

[ref2] Busing F. M. T. A. Commandeur J. J. F. Heiser W. J. (1997). PROXSCAL: a multidimensional scaling program for individual differences scaling with constraints. Adv. Stat. Softw. 6, 237–258.

[ref3] Caspary D. M. Ling L. Turner J. G. Hughes L. F. (2008). Inhibitory neurotransmission, plasticity and aging in the mammalian central auditory system. J. Exp. Biol. 211, 1781–1791. doi: 10.1242/jeb.013581, PMID: 18490394 PMC2409121

[ref4] Conley R. A. Keilson S. E. (1995). Rate representation and discriminability of second formant frequencies for /epsilon/−like steady-state vowels in cat auditory nerve. J. Acoust. Soc. Am. 98, 3223–3234. doi: 10.1121/1.413812, PMID: 8550947

[ref5] Dalton D. S. Cruickshanks K. J. Klein B. E. Klein R. Wiley T. L. Nondahl D. M. (2003). The impact of hearing loss on quality of life in older adults. Gerontologist 43, 661–668. doi: 10.1093/geront/43.5.661 14570962

[ref6] Delgutte B. Kiang N. Y. (1984a). Speech coding in the auditory nerve: V. Vowels in background noise. J. Acoust. Soc. Am. 75, 908–918. doi: 10.1121/1.390537 6707320

[ref7] Delgutte B. Kiang N. Y. (1984b). Speech coding in the auditory nerve: I. Vowel-like sounds. J. Acoust Soc. Am. 75, 866–878. doi: 10.1121/1.390596, PMID: 6707316

[ref8] DePaolis R. A. Janota C. P. Frank T. (1996). Frequency importance functions for words, sentences, and continuous discourse. J. Speech Hear. Res. 39, 714–723. doi: 10.1044/jshr.3904.714 8844552

[ref9] Diehl R. L. (2008). Acoustic and auditory phonetics: the adaptive design of speech sound systems. Philos. Trans. R. Soc. Lond. Ser. B Biol. Sci. 363, 965–978. doi: 10.1098/rstb.2007.2153, PMID: 17827108 PMC2606790

[ref10] Dreschler W. A. Verschuure H. Ludvigsen C. Westermann S. (2001). ICRA noises: artificial noise signals with speech-like spectral and temporal properties for hearing instrument assessment. Int. Coll. Rehabil. Audiol. 40, 148–157. doi: 10.3109/00206090109073110 11465297

[ref11] Dubno J. R. Dirks D. D. Morgan D. E. (1984). Effects of age and mild hearing loss on speech recognition in noise. J. Acoust. Soc. Am. 76, 87–96. doi: 10.1121/1.391011 6747116

[ref12] Fogerty D. Ahlstrom J. B. Bologna W. J. Dubno J. R. (2015). Sentence intelligibility during segmental interruption and masking by speech-modulated noise: effects of age and hearing loss. J. Acoust. Soc. Am. 137, 3487–3501. doi: 10.1121/1.4921603, PMID: 26093436 PMC4474944

[ref13] Fogerty D. Kewley-Port D. Humes L. E. (2012). The relative importance of consonant and vowel segments to the recognition of words and sentences: effects of age and hearing loss. J. Acoust. Soc. Am. 132, 1667–1678. doi: 10.1121/1.4739463, PMID: 22978895 PMC3460985

[ref14] Frisina R. D. Walton J. P. (2006). Age-related structural and functional changes in the cochlear nucleus. Hear. Res. 216-217, 216–223. doi: 10.1016/j.heares.2006.02.003, PMID: 16597491

[ref15] Füllgrabe C. Moore B. C. J. Stone M. A. (2015). Age-group differences in speech identification despite matched audiometrically normal hearing: contributions from auditory temporal processing and cognition. Front. Aging Neurosci. 6:347. doi: 10.3389/fnagi.2014.00347 25628563 PMC4292733

[ref16] Geisler C. D. (1989). The responses of models of "high-spontaneous" auditory-nerve fibers in a damaged cochlea to speech syllables in noise. J. Acoust. Soc. Am. 86, 2192–2205. doi: 10.1121/1.398480, PMID: 2600310

[ref17] Goldberg J. M. Brown P. B. (1969). Response of binaural neurons of dog superior olivary complex to dichotic tonal stimuli: some physiological mechanisms of sound localization. J. Neurophysiol. 32, 613–636. doi: 10.1152/jn.1969.32.4.613, PMID: 5810617

[ref18] Heeringa A. N. Köppl C. (2019). The aging cochlea: towards unraveling the functional contributions of strial dysfunction and synaptopathy. Hear. Res. 376, 111–124. doi: 10.1016/j.heares.2019.02.015, PMID: 30862414

[ref19] Heeringa A. N. Köppl C. (2022). Auditory nerve fiber discrimination and representation of naturally-spoken vowels in noise. eNeuro 9, ENEURO.0474–ENEU21.2021. doi: 10.1523/ENEURO.0474-21.2021 35086866 PMC8856707

[ref20] Heeringa A. N. Teske F. Ashida G. Köppl C. (2023). Cochlear aging disrupts the correlation between spontaneous rate and sound-level coding in auditory nerve fibers. J. Neurophysiol. 130, 736–750. doi: 10.1152/jn.00090.2023 37584075

[ref21] Heeringa A. N. Zhang L. Ashida G. Beutelmann R. Steenken F. Köppl C. (2020). Temporal coding of single auditory nerve fibers is not degraded in aging. J. Neurosci. 40, 343–354. doi: 10.1523/JNEUROSCI.2784-18.2019, PMID: 31719164 PMC6948943

[ref22] Heil P. Neubauer H. Irvine D. R. F. Brown M. (2007). Spontaneous activity of auditory-nerve fibers: insights into stochastic processes at ribbon synapses. J. Neurosci. 27, 8457–8474. doi: 10.1523/JNEUROSCI.1512-07.2007 17670993 PMC6673073

[ref23] Hellstrom L. I. Schmiedt R. A. (1996). Measures of tuning and suppression in single-fiber and whole-nerve responses in young and quiet-aged gerbils. J. Acoust. Soc. Am. 100, 3275–3285. doi: 10.1121/1.417211 8914310

[ref24] Henry K. S. Kale S. Heinz M. G. (2016). Distorted tonotopic coding of temporal envelope and fine structure with noise-induced hearing loss. J. Neurosci. 36, 2227–2237. doi: 10.1523/JNEUROSCI.3944-15.2016, PMID: 26888932 PMC4756156

[ref25] Henry K. S. Sayles M. Hickox A. E. Heinz M. G. (2019). Divergent auditory nerve encoding deficits between two common etiologies of sensorineural hearing loss. J. Neurosci. 39, 6879–6887. doi: 10.1523/JNEUROSCI.0038-19.2019, PMID: 31285299 PMC6733559

[ref210] Huet A. Batrel C. Tang Y. Desmadryl G. Wang J. Puel J. -L. (2016). Sound coding in the auditory nerve of gerbils. Hear Res. 338, 32–39., PMID: 27220483 10.1016/j.heares.2016.05.006

[ref26] Joris P. X. Louage D. H. Cardoen L. van der Heijden M. (2006). Correlation index: a new metric to quantify temporal coding. Hear. Res. 216-217, 19–30. doi: 10.1016/j.heares.2006.03.010, PMID: 16644160

[ref27] Joris P. X. Yin T. C. (1992). Responses to amplitude-modulated tones in the auditory nerve of the cat. J. Acoust. Soc. Am. 91, 215–232. doi: 10.1121/1.402757, PMID: 1737873

[ref28] Jüchter C. Beutelmann R. Klump G. M. (2022). Speech sound discrimination by Mongolian gerbils. Hear. Res. 418:108472. doi: 10.1016/j.heares.2022.108472, PMID: 35276418

[ref29] Kessler D. Carr C. E. Kretzberg J. Ashida G. (2021). Theoretical relationship between two measures of spike synchrony: correlation index and vector strength. Front. Neurosci. 15:761826. doi: 10.3389/fnins.2021.761826, PMID: 34987357 PMC8721039

[ref30] Kessler M. Mamach M. Beutelmann R. Lukacevic M. Eilert S. Bascuñana P.. (2020). GABA_A_ receptors in the Mongolian gerbil: a PET study using [^18^F]flumazenil to determine receptor binding in young and old animals. Mol. Imaging Biol. 22, 335–347. doi: 10.1007/s11307-019-01371-0, PMID: 31102039

[ref31] Khouri L. Lesica N. A. Grothe B. (2011). Impaired auditory temporal selectivity in the inferior colliculus of aged Mongolian gerbils. J. Neurosci. 31, 9958–9970. doi: 10.1523/JNEUROSCI.4509-10.2011, PMID: 21734287 PMC6703306

[ref32] Liberman M. C. Dodds L. W. (1984). Single-neuron labeling and chronic cochlear pathology. II. Stereocilia damage and alterations of spontaneous discharge rates. Hear. Res. 16, 43–53. doi: 10.1016/0378-5955(84)90024-8 6511672

[ref33] Lin F. R. Thorpe R. Gordon-Salant S. Ferrucci L. (2011). Hearing loss prevalence and risk factors among older adults in the United States. J. Gerontol. A Biol. Sci. Med. Sci. 66, 582–590. doi: 10.1093/gerona/glr002, PMID: 21357188 PMC3074958

[ref34] Louage D. H. van der Heijden M. Joris P. X. (2004). Temporal properties of responses to broadband noise in the auditory nerve. J. Neurophysiol. 91, 2051–2065. doi: 10.1152/jn.00816.2003 15069097

[ref35] Macmillan N. A. Creelman C. D. (2004). Detection theory: A User’s guide. 2nd Edn. Psychology Press, New York, NJ.

[ref36] Mardia K. V. Jupp P. E. (2000). Directional statistics. New York, NY: Wiley.

[ref37] Meyer B. T. Jürgens T. Wesker T. Brand T. Kollmeier B. (2010). Human phoneme recognition depending on speech-intrinsic variability. J. Acoust. Soc. Am. 128, 3126–3141. doi: 10.1121/1.3493450, PMID: 21110608

[ref38] Miller R. L. Schilling J. R. Franck K. R. Young E. D. (1997). Effects of acoustic trauma on the representation of the vowel "eh" in cat auditory nerve fibers. J. Acoust. Soc. Am. 101, 3602–3616. doi: 10.1121/1.418321 9193048

[ref39] Moissl U. Meyer-Base U. (2000). A comparison of different methods to assess phase-locking in auditory neurons. Proceedings of the 22nd annual international conference of the IEEE engineering in medicine and biology society, Chicago, IL. 840–843.

[ref40] Parida S. Heinz M. G. (2022a). Underlying neural mechanisms of degraded speech intelligibility following noise-induced hearing loss: the importance of distorted tonotopy. Hear. Res. 426:108586. doi: 10.1016/j.heares.2022.108586 35953357 PMC11149709

[ref41] Parida S. Heinz M. G. (2022b). Distorted tonotopy severely degrades neural representations of connected speech in noise following acoustic trauma. J. Neurosci. 42, 1477–1490. doi: 10.1523/JNEUROSCI.1268-21.2021 34983817 PMC8883846

[ref42] Parthasarathy A. Herrmann B. Bartlett E. L. (2019). Aging alters envelope representations of speech-like sounds in the inferior colliculus. Neurobiol. Aging 73, 30–40. doi: 10.1016/j.neurobiolaging.2018.08.023 30316050 PMC6251750

[ref43] Peterson G. E. Barney H. L. (1952). Control methods used in a study of vowels. J. Acoust. Soc. Am. 24, 175–184. doi: 10.1121/1.1906875

[ref44] Radtke-Schuller S. Seeler S. Grothe B. (2015). Restricted loss of olivocochlear but not vestibular efferent neurons in the senescent gerbil (*Meriones unguiculatus*). Front. Aging Neurosci. 7:4. doi: 10.3389/fnagi.2015.00004 25762929 PMC4327622

[ref45] Ripley S. Xia L. Zhang Z. Aiken S. J. Wang J. (2022). Animal-to-human translation difficulties and problems with proposed coding-in-noise deficits in noise-induced synaptopathy and hidden hearing loss. Front. Neurosci. 16:893542. doi: 10.3389/fnins.2022.893542, PMID: 35720689 PMC9199355

[ref46] Ryan A. (1976). Hearing sensitivity of the mongolian gerbil, Meriones unguiculatis. J. Acoust. Soc. Am. 59, 1222–1226. doi: 10.1121/1.380961, PMID: 956517

[ref47] Sachs M. B. Voigt H. F. Young E. D. (1983). Auditory nerve representation of vowels in background noise. J. Neurophysiol. 50, 27–45. doi: 10.1152/jn.1983.50.1.27 6875649

[ref48] Sachs M. B. Young E. D. (1980). Effects of nonlinearities on speech encoding in the auditory nerve. J. Acoust. Soc. Am. 68, 858–875. doi: 10.1121/1.384825, PMID: 7419821

[ref49] Schmiedt R. A. (1989). Spontaneous rates, thresholds and tuning of auditory-nerve fibers in the gerbil: comparisons to cat data. Hear. Res. 42, 23–35. doi: 10.1016/0378-5955(89)90115-9 2584157

[ref50] Schmiedt R. A. (1996). Effects of aging on potassium homeostasis and the endocochlear potential in the gerbil cochlea. Hear. Res. 102, 125–132. doi: 10.1016/S0378-5955(96)00154-2, PMID: 8951457

[ref51] Schmiedt R. A. (2010). “The physiology of cochlear presbycusis” in The aging auditory system. eds. Gordon-Salant S. Frisina R. D. Popper A. N. Fay R. R., *vol.* 34 (Berlin: Springer), 9–38.

[ref52] Schmiedt R. A. Lang H. Okamura H. O. Schulte B. A. (2002). Effects of furosemide applied chronically to the round window: a model of metabolic presbyacusis. J. Neurosci. 22, 9643–9650. doi: 10.1523/JNEUROSCI.22-21-09643.2002, PMID: 12417690 PMC6758027

[ref53] Schmiedt R. A. Mills J. H. Boettcher F. A. (1996). Age-related loss of activity of auditory-nerve fibers. J. Neurophysiol. 76, 2799–2803. doi: 10.1152/jn.1996.76.4.2799 8899648

[ref54] Sinnott J. M. Mosteller K. W. (2001). A comparative assessment of speech sound discrimination in the Mongolian gerbil. J. Acoust. Soc. Am. 110, 1729–1732. doi: 10.1121/1.1398055 11681351

[ref55] Sinnott J. M. Street S. L. Mosteller K. W. Williamson T. L. (1997). Behavioral measures of vowel sensitivity in Mongolian gerbils (*Meriones unguiculatus*): effects of age and genetic origin. Hear. Res. 112, 235–246. doi: 10.1016/S0378-5955(97)00125-1 9367244

[ref56] Steenken F. Heeringa A. N. Beutelmann R. Zhang L. Bovee S. Klump G. M.. (2021). Age-related decline in cochlear ribbon synapses and its relation to different metrics of auditory-nerve activity. Neurobiol. Aging 108, 133–145. doi: 10.1016/j.neurobiolaging.2021.08.019 34601244

[ref57] Suthakar K. Liberman M. C. (2019). A simple algorithm for objective threshold determination of auditory brainstem responses. Hear. Res. 381:107782. doi: 10.1016/j.heares.2019.107782, PMID: 31437652 PMC6726521

[ref58] Tarnowski B. I. Schmiedt R. A. Hellstrom L. I. Lee F. S. Adams J. C. (1991). Age-related changes in cochleas of Mongolian gerbils. Hear. Res. 54, 123–134. doi: 10.1016/0378-5955(91)90142-V, PMID: 1917712

[ref59] Versteegh C. P. C. Meenderink S. W. F. van der Heijden M. (2011). Response characteristics in the apex of the gerbil cochlea studied through auditory nerve recordings. J. Assoc. Res. Otolaryngol. 12, 301–316. doi: 10.1007/s10162-010-0255-y, PMID: 21213012 PMC3085685

[ref60] Winter I. M. Robertson D. Yates G. K. (1990). Diversity of characteristic frequency rate-intensity functions in guinea pig auditory nerve fibers. Hear. Res. 45, 191–202. doi: 10.1016/0378-5955(90)90120-E, PMID: 2358413

[ref61] Wong J. C. Miller R. L. Calhoun B. M. Sachs M. B. Young E. D. (1998). Effects of high sound levels on responses to the vowel /ε/ in cat auditory nerve. Hear. Res. 123, 61–77. doi: 10.1016/S0378-5955(98)00098-7, PMID: 9745956

[ref62] Wu P. Z. O'Malley J. T. de Gruttola V. Liberman M. C. (2020). Age-related hearing loss is dominated by damage to inner ear sensory cells, not the cellular battery that powers them. J. Neurosci. 40, 6357–6366. doi: 10.1523/JNEUROSCI.0937-20.2020, PMID: 32690619 PMC7424870

[ref63] Young E. D. Sachs M. B. (1979). Representation of steady-state vowels in the temporal aspects of the discharge patterns of populations of auditory-nerve fibers. J. Acoust. Soc. Am. 66, 1381–1403. doi: 10.1121/1.383532 500976

