# Supplementary material for: Altered neural encoding of vowels in noise does not affect behavioral vowel discrimination in gerbils with age-related hearing loss
Source: Front Neurosci. 2023 Nov 14;17:1238941. doi: 10.3389/fnins.2023.1238941 (PMC10682387; doi:10.3389/fnins.2023.1238941)
Supplement: Supplementary file 1 [file Data_Sheet_1.docx]

Supplementary Material

Altered neural encoding of vowels in noise does not affect behavioral vowel discrimination in gerbils with age-related hearing loss

Amarins N. Heeringa^*^, Carolin Jüchter, Rainer Beutelmann, Georg M. Klump, Christine Köppl

*** Correspondence:**Amarins N. Heeringa
[amarins.nieske.heeringa@uni-oldenburg.de](mailto:amarins.nieske.heeringa@uni-oldenburg.de)

# Supplementary Figure 1

**Supplementary Figure 1.** Dominant component schemes of AN fibers recorded in young-adult (panels A, C, E) and quiet-aged gerbils (panels B, D, E) in responses to the vowels /aː/ (panels A, B), /eː/ (panels C, D), and /iː/ (panels E, F). Low-SR fibers (SR < 18 spikes/s) are represented by black symbols; high-SR fibers are represented by colored symbols. The fundamental frequency (*f_0_*) and formant frequencies (*f_1_* and *f_2_*) are indicated to the right of the panels. Harmonics of *f_0_* are depicted in horizontal dotted lines. The black solid line indicates F = BF.
